# Estimation of absolute renal uptake with technetium-99m dimercaptosuccinic acid: direct comparison with the radioactivity of nephrectomy specimens

**DOI:** 10.1590/S1516-31802008000300003

**Published:** 2008-05-01

**Authors:** Mariana da Cunha Lopes de Lima, Celso Darío Ramos, Sérgio Quirino Brunetto, Marcelo Lopes de Lima, Ubirajara Ferreira, Elba Cristina Sá Camargo Etchebehere, Allan de Oliveira Santos, Nelson Rodrigues, Edwaldo Eduardo Camargo

**Keywords:** 99mTc-DMSA, Scintigraphy, Nephrectomy, Kidney cortex, Nuclear medicine, DMSA-99mTc, Cintilografia, Nefrectomia, Córtex renal, Medicina nuclear

## Abstract

**CONTEXT AND OBJECTIVE:**

Studies using radionuclides are the most appropriate method for estimating renal function. Dimercaptosuccinic acid chelate labeled with technetium-99m (^99m^Tc-DMSA) is the radiopharmaceutical of choice for high-resolution imaging of the renal cortex and estimation of the functional renal mass. The aim of this study was to evaluate a simplified method for determining the absolute renal uptake (ARU) of ^99m^Tc-DMSA prior to nephrectomy, using the radioactivity counts of nephrectomy specimens as the gold standard.

**DESIGN AND SETTING:**

Prospective study at the Division of Nuclear Medicine, Department of Radiology, Universidade Estadual de Campinas.

**METHODS:**

Seventeen patients (12 females; range 22-82 years old; mean age 50.8 years old) underwent nephrectomy for various reasons. Renal scintigraphy was performed three to four hours after intravenous administration of a mean dose of 188.7 MBq (5.1 mCi) of ^99m^Tc-DMSA, which was done six to 24 hours before surgery. The *in vivo* renal uptake of ^99m^Tc-DMSA was determined using the radioactivity of the syringe before the injection (measured using a dose calibrator) and the images of the syringe and kidneys, obtained from a scintillation camera. After surgery, the reference value for renal uptake of ^99m^Tc-DMSA was determined by measuring the radioactivity of the nephrectomy specimen using the same dose calibrator.

**RESULTS:**

The ARU measurements were very similar to those obtained using the reference method, as determined by linear regression (r-squared = 0.96).

**CONCLUSION:**

ARU estimation using the proposed method before nephrectomy seems to be accurate and feasible for routine use.

## RESUMO

**CONTEXTO E OBJETIVO:**

Os estudos com radionuclídeos são os mais adequados para se estimar a função renal. O ácido dimercaptosuccínico marcado com tecnécio-99m (DMSA-^99m^Tc) é o radiofármaco de escolha para imagens de alta resolução dos rins, permitindo, também, estimar massa de parênquima renal funcionante. O objetivo deste estudo foi avaliar um método mais simples para determinar-se a captação renal absoluta (CRA) de DMSA-^99m^Tc antes de nefrectomias e validá-lo utilizando-se as contagens radioativas das próprias peças de nefrectomia como padrão-ouro.

**TIPO DE ESTUDO E LOCAL:**

Estudo prospectivo, desenvolvido no Serviço de Medicina Nuclear do Departamento de Radiologia da Universidade Estadual de Campinas.

**MÉTODOS:**

Foram estudados 17 pacientes (12 pacientes do sexo feminino, média de idade de 50,8 anos), selecionados para a realização de nefrectomia. A cintilografia renal foi realizada três a quatro horas após a administração venosa de 188,7 MBq de DMSA-^99m^Tc, seis a 24 horas antes da cirurgia. A CRA *in vivo* foi determinada utilizando-se a radioatividade da seringa antes da injeção (medida com um calibrador de dose) e as imagens da seringa e dos rins, obtidas em uma câmara de cintilação. Após a cirurgia, o valor de referência para a CRA foi determinado medindo-se a radioatividade da peça de nefrectomia com o mesmo calibrador de dose.

**RESULTADOS:**

Os valores de CRA foram muito semelhantes àqueles obtidos com o método de referência, conforme foi demonstrado pela análise de regressão linear (r-quadrado = 0,96).

**CONCLUSÃO:**

A estimativa da CRA com o método proposto antes de nefrectomiasparece ser acurado e aplicável ao uso rotineiro.

## INTRODUCTION

Studies using radionuclides are the most appropriate method for estimating renal function, to add functional information to anatomical studies such as ultrasound and X-ray methods. Dimercaptosuccinic acid chelate labeled with technetium-99m (^99m^Tc-DMSA) was introduced in the early 1970s^1,2^ and, ever since, it has been the radiopharmaceutical of choice for high-resolution imaging of the renal cortex and estimation of the functional renal mass.^3^

Several studies have demonstrated excellent correlation between ^99m^Tc-DMSA uptake and creatinine clearance,^4^ para-aminohippuric acid (PAH) clearance^5^ and renal technetium-99m diethylenetriamine pentaacetic acid (^99m^Tc-DTPA) accumulation.^6,7^

Different techniques to measure the absolute uptake of ^99m^Tc-DMSA by the kidneys have been described using planar scintigraphy and single photon emission computed tomography (SPECT), but the methods remain too complex for routine use.^8,9^ Moreover, none of these techniques for *in vivo* quantification of isolated renal ^99m^Tc-DMSA uptake has been compared with *ex vivo* determination of renal uptake.

## OBJECTIVE

The aim of this study was to evaluate a simplified method for determining the absolute renal uptake (ARU) of ^99m^Tc-DMSA, using the radioactive counts from nephrectomy specimens as the reference.

## METHODS

### Type of study

This was a prospective study conducted in a tertiary public institution.

### Patients

Seventeen patients were studied: twelve females and five males, ranging from 22 to 82 years of age (mean 50.8 years). All of these patients had been selected for nephrectomy by means of open or laparoscopic surgery, because of chronic pyelonephritis (eleven patients) or neoplasia (six patients) (Table 1). All patients signed a consent form that had been approved by the Institution's Ethics Committee.

**Table 1.** Characteristics of the patients who underwent nephrectomy

Patient	Gender	Age	Histopathology
1	Female	56	Chronic pyelonephritis
2	Female	66	Chronic pyelonephritis
3	Female	28	Renal angiomyolipoma
4	Male	82	Urothelial carcinoma of the kidney pelvis and atrophy of the renal parenchyma
5	Male	51	Hydronephrosis and chronic pyelonephritis
6	Male	45	Chronic pyelonephritis
7	Female	54	Chronic pyelonephritis and hydronephrosis
8	Female	60	Renal cell carcinoma
9	Female	45	Chronic pyelonephritis and atrophy of the parenchyma
10	Female	38	Chronic pyelonephritis and hydronephrosis
11	Female	76	Renal cell carcinoma
12	Female	29	Hydronephrosis and atrophy of the parenchyma
13	Female	33	Hydronephrosis, xanthogranulomatosis and chronic pyelonephritis
14	Female	34	Chronic pyelonephritis and hydronephrosis
15	Male	67	Transitional cells carcinoma of the pelvis and ureter
16	Female	22	Ureteral duplication, with severe atrophy of the lower pole; hydronephrosis and hydroureter of the upper unit
17	Male	77	Renal cell carcinoma

The weight (in kg) and height (in cm) of all patients were measured for subsequent use in the Tonnensen equations for kidney depth estimation^10^ (see image processing).

### Radiopharmaceutical preparation

The radiopharmaceutical was reconstituted in accordance with the manufacturer's instructions (CIS Bio International DMSA agent, Gif-sur-Yvette, France; IPEN Molybdenum generator [Mo-99-Tc99m], São Paulo, Brazil).

The labeling efficiency was determined by means of thin layer chromatography silica gel (TLC-SG) kits (Merck, Darmstadt, Germany). The syringes with the doses of ^99m^Tc-DMSA were measured in a dose calibrator (Capintec CRC-15R, Ramsey, New Jersey, United States). The doses ranged from 173.9 to 207.2 MBq (4.7 to 5.6 mCi), with a mean dose of 188.7 MBq (5.1 mCi). After intravenous injection, the residual radioactivity of the syringes was also measured. The radioactivity administered to the patients was calculated by subtracting this residual radioactivity from the syringe radioactivity before injection.

### Syringe images

The syringes containing the ^99m^Tc-DMSA dose were imaged before intravenous administration to the patients, to determine the efficiency of the scintillation camera detector. The images were acquired for 120 seconds (matrix 256 x 256, zoom 1.8) in a single-head scintillation camera equipped with a high-resolution collimator (SP4 HR Elscint-General Electric, Haifa, Israel).

To keep the radioactivity measured in the syringes within the linear range of the detector, the syringes were placed inside a lead cylinder during acquisition. This lead cylinder was developed by the Institution's Physics Division and consisted of a cylindrical polyvinyl chloride (PVC) tube of 3.8 cm in diameter by 20.3 cm in length inside a lead sheath of 0.1 cm in thickness. The attenuation correction factor was obtained experimentally, as follows.

### Determination of the lead device attenuation correction factor

Radioactivity counts were acquired ten times for each of five samples of technetium-99m with different radioactivity levels (18.5 MBq; 37 MBq; 74 MBq; 148 MBq; 222 MBq) with the syringes inside the lead cylinder, using the same scintillation camera and acquisition parameters. All acquisitions were then repeated without the lead cylinder.

After decay and background corrections, the mean count for each technetium-99m sample was determined. The data obtained with and without the lead device were plotted on two graphs and the attenuation correction factor for the cylinder was then obtained by dividing the two angular coefficients, resulting in the factor 2.8087.

### Patient images

**Figure 1:**
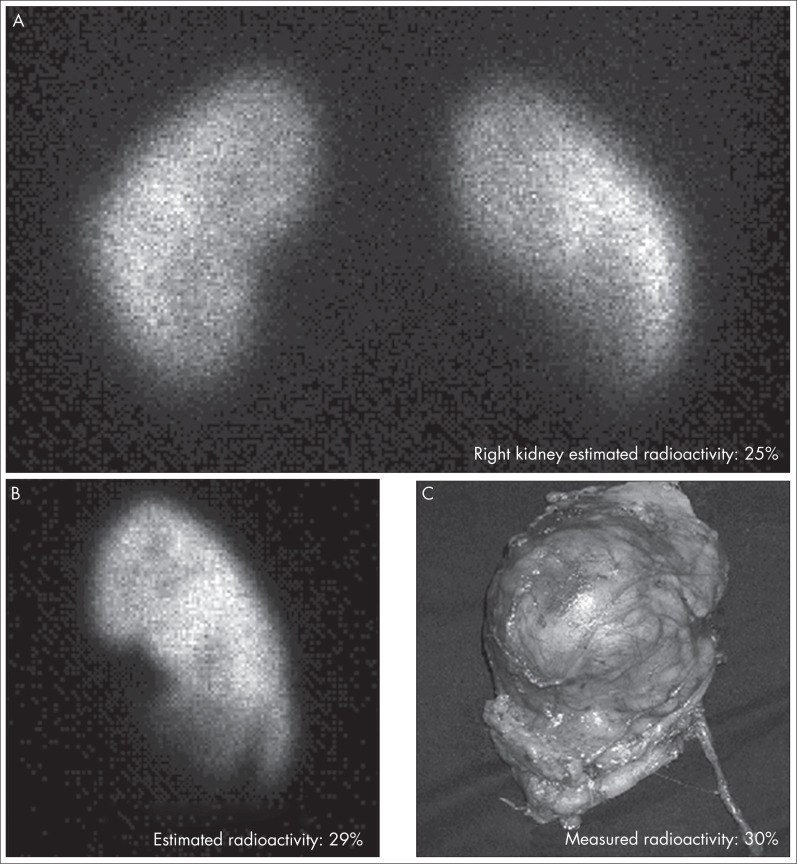
Estimation of absolute renal uptake with technetium-99m dimercaptosuccinic acid (^99m^Tc-DMSA) in a 28-year-old patient with a right kidney tumor diagnosed by computed tomography (a: *in vivo* image, posterior view; b: *ex vivo* image, posterior view; c: nephrectomy specimen, anterior view).

Renal scintigraphy was performed three to four hours after administering the radiopharmaceutical, and six to 24 hours prior to surgery. Posterior view images in the supine position (matrix 256 x 256; zoom 1.8) were acquired with 800,000 counts, using the same camera as used for syringe imaging (Figure 1A). Tracer extravasation to soft tissue during injection did not occur in any of the patients, as demonstrated by imaging each patient's injection site.

The same couch and position used to acquire the patient images were also used to acquire the syringe image, in order to standardize the attenuation factor.

### Image processing

#### Determination of renal counts per second

Using the isocontour method (30%), regions of interest (ROIs) were drawn over the kidneys that would be removed. All ROIs were visually checked for accuracy. In four cases, the ROI was not properly drawn by the isocontour method, and a new ROI was manually drawn. The number of counts per second in the kidneys was determined. Background corrections were performed using ROIs around the kidneys. All values were corrected for radioactive decay.

#### Estimation of kidney depth:

The depth of the kidneys in centimeters was estimated by the Tonnensen equations,^10^ based on the weight (W) in kg and height (H) in cm of each patient, as follows:


$$\begin{array}{l} \text{Right kidney}=13.3\;\left(\text{W}/\text{H}\right)+0.7\\ \text{Left kidney}=13.2\;\left(\text{W}/\text{H}\right)+0.7 \end{array}$$

#### Kidney attenuation correction and determination of the renal corrected counts per second:

Tissue attenuation corrections were performed by taking into consideration the attenuation of technetium-99m photons in water (0.15),^11^ which is the most similar analogy to human tissue. The following equation was used:


$$\text{Renal corrected counts per second}=\frac{\text{Renal counts per second}}{{\text{e}}^{-\left(0.15\right)\text{x}\left(\text{kidney depth}\right)}}$$

#### Determination of syringe counts per second:

The count per second was obtained from the syringe images, with correction for radioactive decay, and multiplication by the attenuation factor (2.8087).

### Estimation of the ARU of ^99m^Tc-DMSA using the images

The estimation of the ARU was based on the efficiency of the detector (counts/MBq x seconds), by dividing the syringe count per second before injection by the radioactivity of the same syringe measured by the dose calibrator.


$$\text{Efficiency of the detector}=\frac{\text{Syringe count per second before injection}}{\text{Radioactivity of the syringe before injection}}$$

The ARU was then calculated using the images of the kidneys before nephrectomy:


$$\text{ARU}\frac{\text{Renal corrected count per second}/\text{efficiency}}{\text{Radioactivity administered to the patient}}\text{x}100$$

### ARU using nephrectomy specimen images (ex vivo renal images) for evaluating the attenuation correction accuracy

Immediately after surgery, posterior view images of the nephrectomy specimens were obtained using the same scintillation camera as before, with the same acquisition parameters (Figure 1B). These images were not subjected to soft tissue attenuation (except for the attenuation of the renal parenchyma itself).

All ARU values were then recalculated using these images and the equation described above. These values were compared with the values obtained from the *in vivo* images and with the reference value.

### Determination of the reference value for the ARU of ^99m^Tc-DMSA

Each nephrectomy specimen was placed in a plastic bag and fitted in the same dose calibrator chamber that had been used for radioactivity measurement on the syringes. After correcting all measurements for radioactive decay, the reference value for the ARU was determined for each excised kidney (Figure 1C) as a percentage of the injected dose, by dividing the nephrectomy specimen radioactivity by the injected dose radioactivity and multiplying by 100%, as follows:


Reference valur of ARU=(specimen radioactivity/injected radioactivity)x100%

### Statistical analysis

Linear regression analysis was used to compare the ARU calculated using *in vivo* and *ex vivo* images with the ARU from the reference method. It was also applied to compare the ARU values measured with the *in vivo* and *ex vivo* images in order to evaluate the similarity of these values, which is related to the accuracy of the attenuation correction method applied to the *in vivo* images of kidneys.

## Results

The labeling efficiency of the radiopharmaceutical ranged from 98.7% to 99.3%. The mean ARU values obtained with the *in vivo* and *ex vivo* images and with the reference method were, respectively, 5.6%, 6.4% and 6.8%. The calculated ARU values for all patients are listed in Table 2.

**Table 2.** Absolute renal uptake (ARU) of technetium-99m dimercaptosuccinic acid (^99m^Tc-DMSA): comparison with the reference value

Patient	ARU, *in vivo* image (%)	ARU, *ex vivo* image (%)	Reference (nephrectomy specimen radioactivity) (%)
1	3.2	3.9	3.8
2	0.9	0.9	1.0
3	24.6	27.8	30.3
4	2.5	6.7	7.3
5	3.6	2.8	3.1
6	3.1	2.1	2.6
7	6.0	7.3	7.7
8	14.1	14.7	17.0
9	0.3	0.4	0.4
10	2.3	2.9	2.9
11	8.3	8.1	9.0
12	5.7	8.1	6.1
13	0.6	1.4	1.7
14	6.0	5.4	5.8
15	0.9	0.5	0.6
16	4.9	4.5	4.9
17	8.5	11.9	12.3

**Figure 2:**
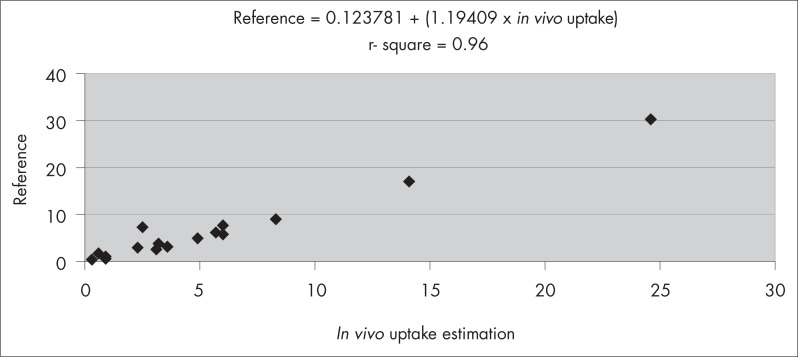
Linear regression analysis between the reference method and the *in vivo* absolute renal uptake of technetium-99m dimercaptosuccinic acid (^99m^Tc-DMSA).

**Figure 3:**
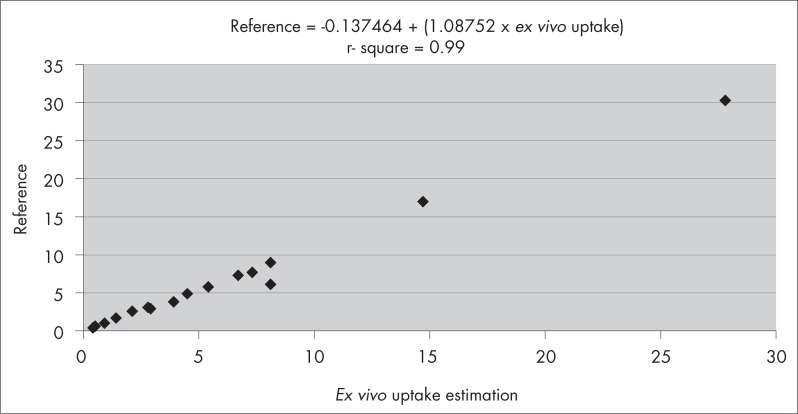
Linear regression analysis between the reference method and the *ex vivo* absolute renal uptake of technetium-99m dimercaptosuccinic acid (^99m^Tc-DMSA).

The linear regression analysis comparing the ARU values obtained with the *in vivo* and *ex vivo* images and the reference method resulted in *r*-squared of 0.96 (*in vivo* image and the reference method, Figure 2) and 0.99 (*ex vivo* image and the reference method; Figure 3).

The attenuation correction method applied to the *in vivo* images was found to be efficient, since the values obtained with the *in vivo* and *ex vivo* images were very similar, with *r*-squared of 0.95.

## DISCUSSION

Determination of the functional capability of an individual kidney is particularly important when nephrectomy is being considered. Many studies have evaluated different methods for quantifying ^99m^Tc-DMSA uptake.^8,9,12^ However, few studies have specifically addressed the use of the ARU measurement to evaluate the kidneys for nephrectomy purposes.^13,14^ When surgery is considered, very precise measurement of the absolute renal function is important, since chronic renal failure after nephrectomy is a possibility.^13^ In addition, we have observed that nephrectomy provides a good opportunity to check the efficiency of the method used for absolute renal uptake estimation using ^99m^Tc-DMSA.

Goldraich et al.^15^ also determined the absolute renal uptake in 142 children with vesicoureteral reflux based on the percentage of the injected dose of ^99m^Tc-DMSA. Their technique was similar to that described by Raynaud^16^ using ^197^Hg-chlormerodrin (^197^HgCl_2_), which was an innovative quantitative method at that time. Goldraich et al.^15^ reported a significant association between the degree of reflux nephropathy and the functional impairment measured by ^99m^Tc-DMSA uptake. Their technique needed a correction factor consisting of standard radioactivity for renal depth, calculated according to a decreasing exponential curve that was obtained by plotting a series of measurements of the standard against increasing thicknesses of a Plexiglas^®^ cover.

Morris et al.^17^ measured the absolute renal uptake of ^99m^Tc-DMSA in 160 children, based on injected counts, by subtracting the count in the syringe after injection from the count before injection. The attenuation correction used the geometric mean count between the anterior and posterior positions. They considered the thickness of the patient's intervening tissue at the center of each kidney, measured with the patients in the prone and supine positions. They determined the count in the syringe before injection by acquiring an image for only five seconds, in order to avoid pixel overflow in the computer. In the present study, we preferred to use a lead cylinder attenuator.

Groshar et al. proposed the use of a quantitative SPECT method to estimate the absolute renal function,^12^ based on the technique of Iosilevsky et al.^18^ The latter is a very sophisticated method that avoids the need for renal depth determination. However, it is laborious and impractical, and depends on specific site standardizations, including the need to determine the threshold value for each institution. Groshar et al.^12^ did not use a gold standard test to confirm their final results.

In the present method, we performed attenuation correction by using the renal depth estimated by the Tonnensen equations.^10^ which are simple for routine use and have been used by several authors.^19,20^ These equations may underestimate renal depth, as previously described by Taylor et al.^21^ Nevertheless, we were able to check the efficiency of this method by comparing the ARU measurements of the *in vivo* and *ex vivo* images, which turned out to be very similar, with excellent correlation (r-squared = 0.95).

The use of the cylindrical device made of lead and PVC was essential for keeping the radioactivity dose within the linear range of the scintillation camera detector, with correction of the error due to dead time loss. A device of this nature can be easily developed in any institution.

In this study, we evaluated a simple method for determining the ARU before surgery using a reliable gold standard, which consisted of direct measurement of renal radioactivity in the nephrectomy specimen. The ARU values obtained were very similar to those of the reference method, with r-squared of 0.96. Using simple parameters, namely the radioactivity and count in the syringe and the count in the kidney, this method is easy to introduce into routine clinical practice. In particular, measurement of the radioactivity and acquisition of an image of the syringe inside the lead device is necessary only once a day. Thus, the only parameters needed for each patient are the injected dose and the count in the kidney.

It is important to emphasize that the method proposed here was only performed on entopic kidneys that were being considered for nephrectomy, mostly with severely reduced function. Further studies are necessary in order to evaluate whether this method would be feasible for ectopic organs and kidneys with normal or mildly impaired function.

The optimal time at which to determine the absolute renal uptake of ^99m^Tc-DMSA has been reported to be six hours after injection. By that time, it is postulated that the uptake will have reached a plateau.^22^ However, for ideal imaging characteristics, a time interval of 2-4 hours between injection and imaging is optimal, without any significant effect on absolute ^99m^Tc-DMSA uptake.^23^ The difference between the values obtained four hours and six hours after injection is less than 6%.^24^

The fact that the *in vivo* images were acquired three to four hours after administering the radiopharmaceutical, while the images of the nephrectomy specimens were acquired six to 24 hours later could represent a potential error in the *in vivo* ARU values, compared with the specimen ARU values. Nevertheless, we found a high correlation between the *in vivo* and specimen ARU values (r*-*squared = 0.96), which suggests that there was no significant change in the renal uptake over this interval of time, at least for this group of patients, whose kidneys mainly presented low functional capability.

Thus, in the present study, a simplified method was used to quantify kidneys that were scheduled for nephrectomy. The *in vivo* ARU was compared with the gold standard, i.e. the “real” quantification of the functional capability of the resected kidney, which was determined as the percentage of the injected radioactivity present in the nephrectomy specimen.

## CONCLUSIONS

The method described is simple and precise for estimating the ARU before nephrectomy, without the need for imaging phantoms or complex equations. However, further studies are needed, in order to evaluate the usefulness of this method in kidneys with normal or mildly impaired function.

## References

[1] Lin TH, Khentigan A, Winchell HS (1974). A 99mTc-chelate substitute for organoradiomercurial renal agents. J Nucl Med.

[2] Müller-Suur R, Magnusson G, Bois-Svensson I, Jansson B (1991). Estimation of technetium 99m mercaptoacetyltriglycine plasma clearance by use of one single plasma sample. Eur J Nucl Med.

[3] Taylor A, Blaufox MD (1989). Radiopharmaceuticals for the measurement of ‘functional renal mass’. Evaluation of renal function and disease with radionuclides: the upper urinary tract.

[4] Daly MJ, Jones W, Rudd TG, Tremann J (1979). Differential renal function using technetium-99m dimercaptosuccinic acid (DMSA): in vitro correlation. J Nucl Med.

[5] Higashihara E, Tokuda H, Kishi H (1988). Technetium-99m dimercaptosuccinic acid uptake in long-term catheterized kidney. Comparison with renal function. Urology.

[6] Bingham JB, Maisey MN (1978). An evaluation of the use of 99Tcm-dimercaptosuccinic acid (DMSA) as a static renal imaging agent. Br J Radiol.

[7] Taylor A (1982). Quantitation of renal function with static imaging agents. Semin Nucl Med.

[8] Kawamura J, Hosokawa S, Yoshida O (1979). Renal function studies using 99mTc-dimercaptosuccinic acid. Clin Nucl Med.

[9] Kawamura J, Hosokawa S, Yoshida O, Fujita T, Ishii Y, Torizuka K (1978). Validity of 99mTc dimercaptosuccinic acid renal uptake for an assessment for individual kidney function. J Urol.

[10] Tonnensen KH, Munch O, Hald T, Zum Winkel K, Blaufox MD, Funck-Brentano JL (1974). Influence on the renogram of variation skin to kidney distance and the clinical importance hereof. Radionuclides in Nephrology, Proceedings of the Third International Symposium on Radionuclides in Nephrology.

[11] Gates GF (1983). Split renal function testing using Tc-99m DTPA. A rapid technique for determining differential glomerular filtration. Clin Nucl Med.

[12] Groshar D, Moskovitz B, Gorenberg M (1994). Quantitative SPECT of technetium-99m-DMSA uptake in the kidneys of normal children and in kidneys with vesicoureteral reflux: detection of unilateral kidney disease. J Nucl Med.

[13] Mullerad M, Kastin A, Issaq E, Moskovitz B, Groshar D, Nativ O (2003). The value of quantitative 99m technetium dimercaptosuccinic acid renal scintigraphy for predicting postoperative renal insufficiency in patients undergoing nephrectomy. J Urol.

[14] Kawamura J, Itoh H, Okada Y (1983). Preoperative and postoperative cortical function of the kidney with staghorn calculi assessed by 99mtechnetium-dimercaptosuccinic acid renal scintigraphy. J Urol.

[15] Goldraich NP, Goldraich IH, Anselmi OE, Ramos OL (1984). Reflux nephropathy: the clinical picture in South Brazilian children. Contrib Nephrol.

[16] Raynaud C (1974). A technique for the quantitative measurement of the function of each kidney. Semin Nucl Med.

[17] Morris SC, Chittenden SJ, Rivens I, Heary TA, Vanstone C, Meller ST (1995). Absolute 99Tcm-DMSA renal uptake in children: a study of 321 kidneys. Nucl Med Commun.

[18] Iosilevsky G, Israel O, Frenkel A (1989). A practical SPECT technique for quantitation of drug delivery to human tumors and organ absorbed radiation dose. Semin Nucl Med.

[19] Gates GF (1982). Glomerular filtration rate: estimation from fractional renal accumulation of 99mTc-DTPA (stannous). AJR Am J Roentgenol.

[20] Chachati A, Meyers A, Godon JP, Rigo P (1987). Rapid method for the measurement of differential renal function: validation. J Nucl Med.

[21] Taylor A, Lewis C, Giacometti A, Hall EC, Barefield KP (1993). Improved formulas for the estimation of renal depth in adults. J Nucl Med.

[22] Gordon I (1987). Indications for 99mtechnetium dimercapto-succinic acid scan in children. J Urol.

[23] Moorin R (2001). 99mTc-DMSA absolute uptake: normal pediatric values at 2-4 hours. J Nucl Med Technol.

[24] Flower MA, Meller ST, Chittenden SJ, Fielding SL, Evans K, Gordon I (1995). Absolute 99Tcm-DMSA renal uptake in children: optimum time to scan. Nucl Med Commun.

